# Paleopathological Description and Diagnosis of Metastatic Carcinoma in an Early Bronze Age (4588+34 Cal. BP) Forager from the Cis-Baikal Region of Eastern Siberia

**DOI:** 10.1371/journal.pone.0113919

**Published:** 2014-12-03

**Authors:** Angela R. Lieverse, Daniel H. Temple, Vladimir I. Bazaliiskii

**Affiliations:** 1 Department of Archaeology and Anthropology, University of Saskatchewan, Saskatoon, SK, S7N 5B1, Canada; 2 Department of Sociology and Anthropology, George Mason University, Fairfax, Virginia, United States of America; 3 Department of Archaeology and Ethnography, Irkutsk State University, Irkutsk, 664003, Russian Federation; University of Florence, Italy

## Abstract

Extensive osteolytic and osteoblastic lesions were observed on the skeletal remains of an adult male excavated from an Early Bronze Age cemetery dated to 4556+32 years BP, located in the Cis-Baikal region of Siberia (Russian Federation). Lytic lesions ranged in size from several mm to over 60 mm in diameter and had irregular, moth-eaten borders. Many of these lesions destroyed trabecular bone, though a hollowed shell of cortical bone often remained observable. Radiographic analysis revealed numerous lytic lesions within trabecular bone that had not yet affected the cortex. Blastic lesions were identified as spiculated lines, bands, or nodules of mostly immature (woven) bone formed at irregular intervals. Anatomical elements with the greatest involvement included those of the axial skeleton (skull, vertebrae, sacrum, ribs, and sternum) as well as proximal appendicular elements (ossa coxae, proximal femora, clavicles, scapulae, and proximal humeri). Osteocoalescence of destructive foci was observed on the ilium and frontal bone, with the largest lesion found on the right ilium. Differential diagnoses include metastatic carcinoma, mycotic infections, tuberculosis, Langerhan's cell histiocytosis, and multiple myeloma. Based on lesion appearance and distribution, age and sex of the individual, as well as pathogen endemism, the most likely diagnostic option for this set of lesions is metastatic carcinoma. The age and sex of this individual and appearance of the lesions may reflect carcinoma of the lung or, possibly, prostate. This represents one of the earliest cases of metastatic carcinoma worldwide and the oldest case documented thus far from Northeast Asia.

## Introduction

Evidence for neoplasia in antiquity—and malignant neoplasia (cancer) in particular—is relatively scarce despite a growing number of reported cases published in the archaeological and paleopathological literature (e.g., [1]–[14]). This, in contrast to the pervasiveness of the condition today, with cancer being the second leading cause of death in industrialized nations [15], suggests that there remains tremendous gaps in knowledge regarding the history and evolution of neoplastic diseases [16]–[17]. Paleopathological investigation of neoplasia can, however, shed light on a range of these issues, including the distribution and variation of neoplastic diseases across time and space, as well as their relationships with changing demographic, genetic, and environmental factors [16], [18].

Many scholars believe that neoplasia was substantially less common in the past than it is today, predominately reflecting shorter human life spans and more favorable environmental factors [15]–[17], [19]–[22]. Others maintain that the prevalence of ancient malignancies, at least in the last several millennia, was not significantly different than that of modern industrialized societies [23]. This debate notwithstanding, it should come as no surprise that the number of archaeologically documented cases of neoplasia decreases relative to sample size and time depth: neoplasia frequency increases at sites that are relatively more recent and have larger sample sizes [16]. In fact, neoplastic diseases documented on human (or pre-human) skeletal material older than about 4000 years are remarkably rare, most reflecting “possible” neoplasia and/or benign conditions [7], [24]–[30]. While malignant cancers—metastatic carcinoma and multiple myeloma, in particular—are among the most common types of neoplasia documented on archaeological remains [16], [31]–[32], the vast majority of these cases date within the last four millennia [2]–[4], [6], [8]–[13], [33].

Here we present a case of malignant neoplasia from the Early Bronze Age (5200/5000–4000 cal. BP) hunter-gatherer cemetery of Gorodishche II, located in the Cis-Baikal region of Siberia. Extensive osteolytic and osteoblastic lesions located predominately on the axial and proximal elements of the appendicular skeleton indicate metastatic carcinoma, possibly of the lung or prostate. This represents one of the earliest cases of cancer metastasis among hunter-gatherers worldwide and the oldest case documented thus far from Northeast Asia.

## Materials and Methods

This research is part of the Baikal-Hokkaido Archaeology Project (BHAP), housed at the University of Alberta, Canada. The BHAP and University of Alberta have an Agreement of Cooperation with Irkutsk State University (Russian Federation), where the skeletal remains described in this paper are curated. Permission is granted through this agreement (a copy of which was submitted to the PLOS editors for review) for BHAP researchers to access and study the hundreds of middle Holocene—Neolithic through Bronze Age—human remains housed at Irkutsk State University. The specimen discussed in this manuscript (Gorodishche II, Burial 3) was excavated by the third author, Vladimir Ivanovich Bazaliiskii of Irkutsk State University. A copy of Bazaliiskii's excavation permit (#608) from the Russian Academy of Science's Archaeological Institute was also submitted to the editors review. All the human skeletal remains described in this study are curated by Irkutsk State University and were accessed with the permission of our Russian hosts and collaborators. Appropriate permits were obtained and regulations complied with.

**Figure 1:**
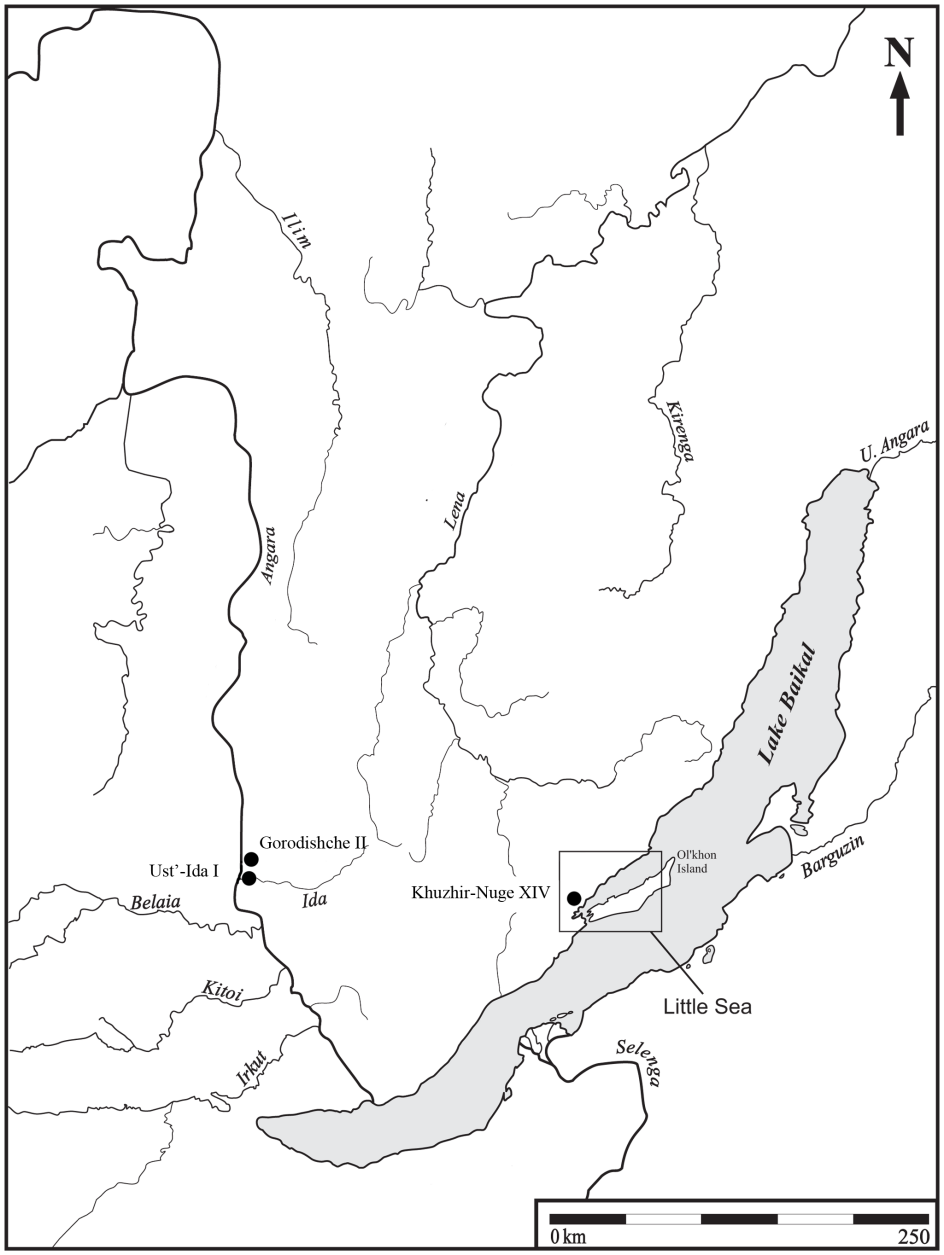
Map of Cis-Baikal with location of the Gorodishche II cemetery.

Gorodishche II is a small Early Bronze Age hunter-gatherer cemetery located in the Cis-Baikal, the vast mountainous region north and west of Lake Baikal in Southeastern Siberia, Russian Federation (Fig. 1). The cemetery is situated on the east bank of the Angara River, about 125 km north of the modern city of Irkutsk and 2.5 km north of the much larger Late Neolithic-Early Bronze Age cemetery of Ust'-Ida I. Frequently, the terms ‘Neolithic’ and ‘Bronze Age’ imply sedentism, agriculture, and metallurgy. However, in Siberian archaeology, Neolithic and Bronze Age economies are characterized by the introduction of pottery, ground stone, and bow and arrow technology, and by the appearance of (mostly decorative) copper and bronze, respectively [34]–[37]. Indeed, the Middle Holocene occupants of the Cis-Baikal were, in every sense of the word, mobile foragers subsisting predominately on game hunting, fishing, and sealing.

**Figure 2:**
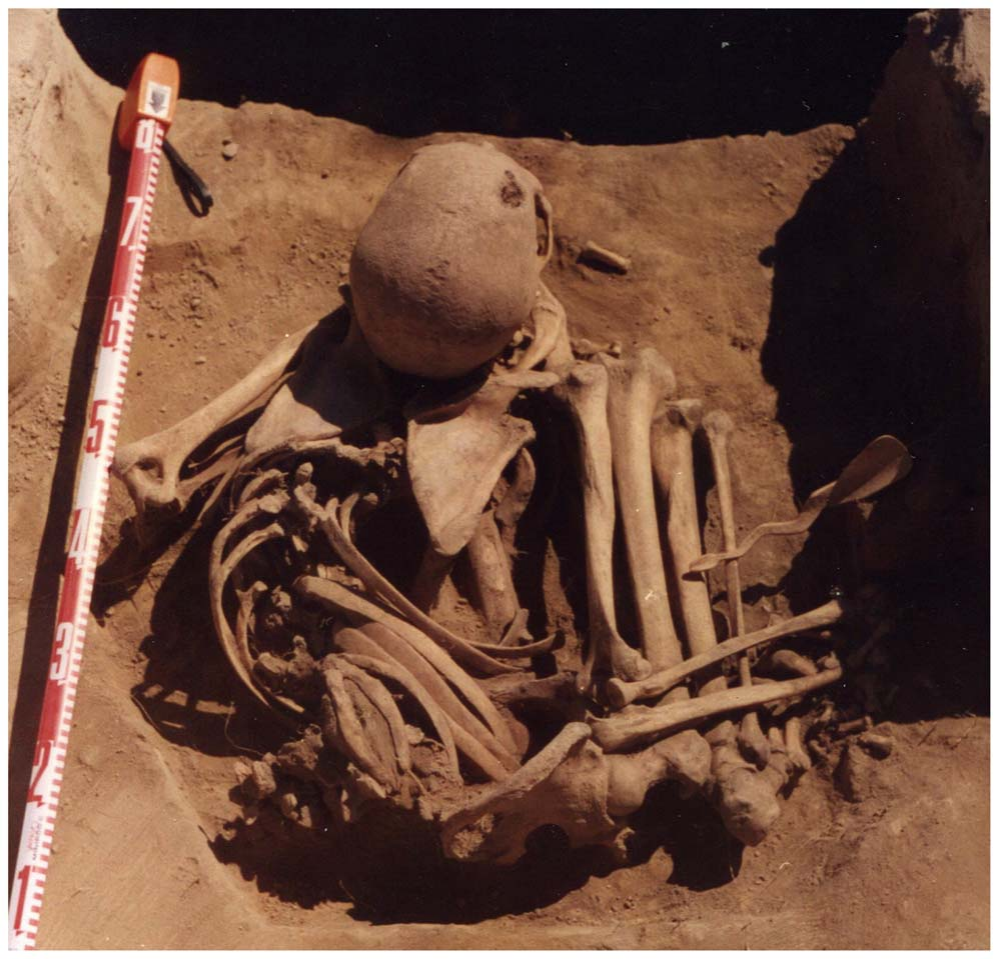
In situ photograph of Gorodishche II, Burial 3. North is to the reader's (and skeleton's) left.

Three single interments were excavated from Gorodishche II in 1997, two dating to the Early Bronze Age (5200/5000–4000 cal. BP) and one to the more recent Middle-Late Bronze Age. A fourth burial was completely destroyed. Burial 3, the subject of this paper, was one of the two Early Bronze Age burials recovered. Like the other interments at the site, it comprised a single individual placed in a pit and covered with a stone cairn. The burial pit was circular, measuring approximately 70 cm in diameter and 70 cm deep. The body was lying in a prone but tightly flexed position, with the knees drawn up to the chest (Fig. 2). Several remarkable grave goods were recovered with the body, including an ornamental bone tubule and an intricately carved bone spoon with a winding serpent handle (Fig. S1). The burial was directly radiocarbon dated to 4588–4524 years BP (OxA-26895: 4556±32 BP).

**Figure 3:**
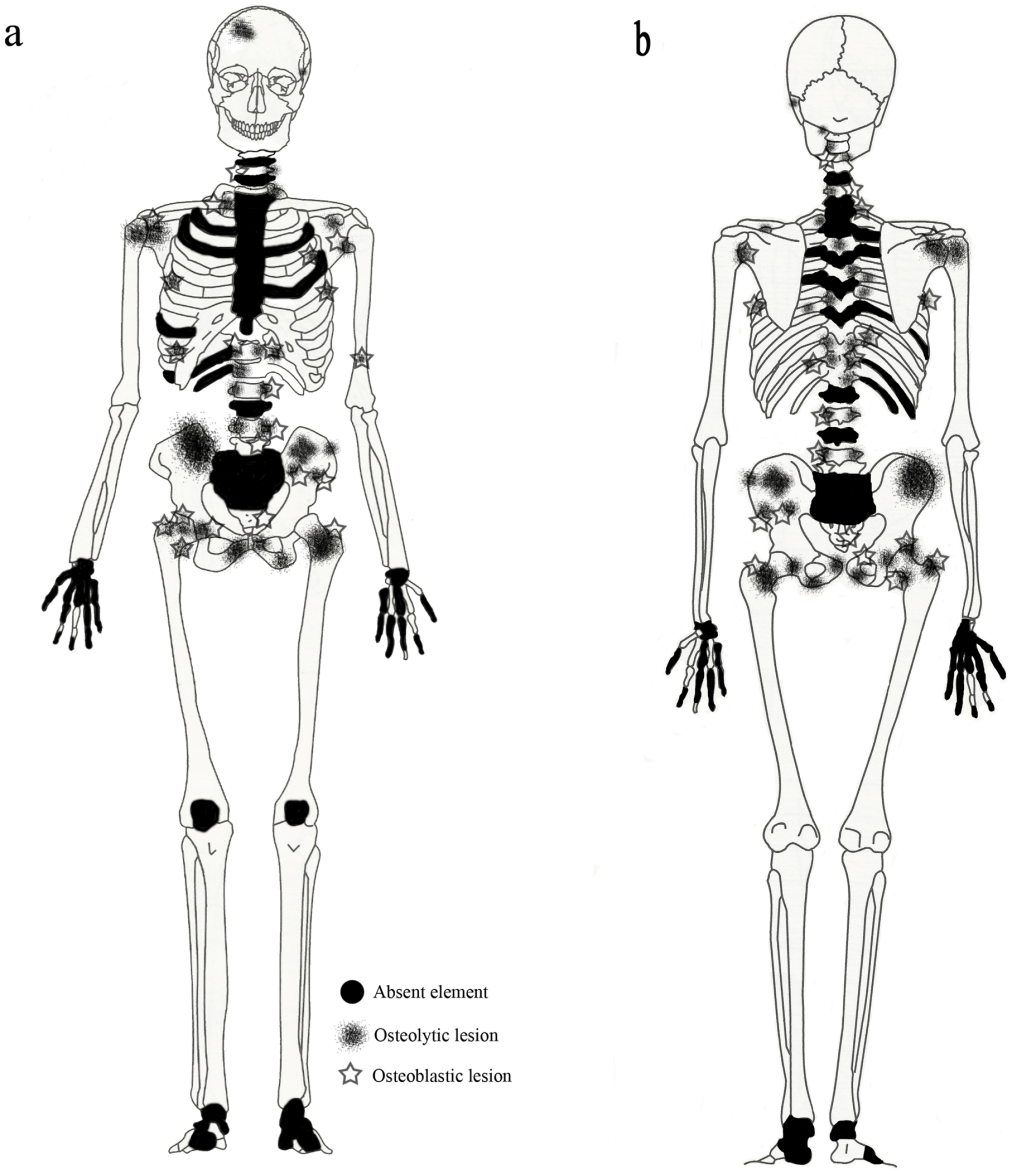
Burial 3: Diagrammatic representation of skeletal completeness and lesion distribution; a, anterior view; b, posterior view.

In 2012, a full osteological assessment of Burial 3 was completed by the first two authors. The skeleton was largely complete and well preserved, including the intact cranium and mandible, all major limb bones (long bones and those of the pectoral and pelvic girdles), most vertebrae and ribs, and about a third of the pedal and manual elements (Fig. 3). A conservative age at death estimate of 35–45 years was based on the morphology of the pubic symphysis and iliac auricular surface, as well as palatal and ectocranial suture closure [38]–[41]. Sex was determined as male based on cranial and pelvic morphology (as outlined by Buikstra and Ubelaker, [42]). Some evidence of dental disease (periodontitis, calculus deposition, and antemortem tooth loss of the right maxillary first and second molars) was observed. However, the most apparent pathological changes involved extensive osteolytic and osteoblastic lesions distributed on most of the axial elements and the proximal appendicular bones (Fig. 3). In a number of cases, large portions of bones were missing altogether, likely the result of osteolytic destruction and/or taphonomic degradation subsequent to osteolysis. In addition to photographic, metric, and descriptive documentation of each lesion, radiographs were taken of the cranium, left ilium, right femur, and right humerus. A diagrammatic representation and complete summary of all documented lesions are presented in Figure 3 and Table 1, respectively.

**Table 1:** Gorodishche II, Burial 3: List of Observed Osseous Lesions.

Lesion	Element Observed	Portion Affected	Type	Size	Coalescence
1	Frontal	R superior squama	Lytic	35	yes, with 2
2	Frontal	R superior squama	Lytic	10	yes, with 1
3	L sphenoid	Temporal surface of greater wing	Lytic	15	no
4	Occipital	Anterior edge of L pars lateralis	Lytic	15	no
5	Axis	L lamina, L inferior articular process, posterior aspect of L superior articular process, L transverse process	Lytic	CD	*
6	C3 vertebra	R superior & inferior articular processes	Lytic	CD	*
7	C3 vertebra	L superior articular facet	Blastic	*	no
8	C6 vertebra	L transverse process, L superior articular process, superior aspect of L inferior articular process	Lytic	CD	*
9	C6 vertebra	R inferior articular facet	Blastic	*	no
10	T vertebra A	L posteroinferior centrum	Lytic	20	no
11	T vertebra B	tip of R transverse process	Lytic	17	no
12	T vertebra B	L neural arch, spinous process, medial aspect of R lamina	Lytic	CD	*
13	T vertebra C	R transverse process	Lytic	CD	no
14	T vertebra C	R superior centrum	Lytic	30	no
15	T vertebra D	tip of R transverse process	Mixed	10	yes
16	T vertebra D	centrum, L pedicle, anterior surface of L superior articular process, anterior R pedicle	Lytic	CD	*
17	T vertebra E	anterior spinous process	Lytic	*	*
18	T12 vertebra	R posterior centrum, R pedicle, R transverse process, R superior & inferior articular processes	Lytic	CD	*
19	L vertebra	L superior articular process	Lytic	10	no
20	L vertebra	centrum, R pedicle, R transverse process, R superior articular process	Lytic	CD	*
21	L vertebra	posterior L lamina	Mixed	10	no
22	L4 vertebra	R superoposterior centrum	Lytic	12	no
23	L4 vertebra	L inferior articular process	Blastic	*	no
24	L4 vertebra	L lamina	Mixed	10	no
25	L5 vert fragment	L posterior centrum	Blastic	*	*
26	L vert fragment A	inferior aspect of superior articular facet	Blastic	*	*
27	L vert fragment B	anterior L lamina, posterior L pedicle	Mixed	*	*
28	sacrum, inferior third	anterior surface	Blastic	*	*
29	L 1st rib	head, neck, tubercle	Lytic	CD	*
30	L 11th rib	proximal shaft	Mixed	15	no
31	L rib A	midshaft, pleural surface	Mixed	25	no
32	L rib B	head, neck, tubercle	Lytic	CD	*
33	L rib fragment	midshaft	Mixed	*	*
34	R rib A	head, neck, tubercle	Lytic	CD	*
35	R rib A	inferior border of midshaft	Mixed	22	no
36	R rib B	head, neck, tubercle	Lytic	CD	*
37	R rib C	head, neck, tubercle, proximal end	Mixed	CD	*
38	R rib fragment	sternal end	Mixed	CD	*
39	R clavicle	1–2 cm of sternal end	Mixed	CD	*
40	L scapula	base of coracoid process	Lytic	8.5	no
41	L scapula	inferioanterior glenoid fossa & adjacent neck	Mixed	17	no
42	R scapula	inferior coracoid process, glenoid fossa, neck	Mixed	CD	*
43	L humerus	anterior distal shaft	Mixed	9	no
44	R humerus	posterior head	Lytic	19	no
45	L os coxae	posterior half of iliac blade, including superior auricular surface	Lytic	66	yes, with 46
46	L os coxae	posterior iliac body	Mixed	30	yes, with 45
47	L os coxae	anteroinferior iliac blade	Mixed	41	yes
48	L os coxae	mid iliac crest	Lytic	30	no
49	L os coxae	posterinferior acetabulum and superior ischium	Lytic	CD	*
50	L os coxae	posterior ischiopubic ramus	Lytic	CD	*
51	L os coxae	pubic body	Lytic	20	no
52	R os coxae	posterior third of iliac blade, including iliac tuberosity, posterior superior iliac spine, and posterior third of iliac crest	Lytic	CD	*
53	R os coxae	anterosuperior auricular surface	Lytic	15	no
54	R os coxae	anterior acetabulum and posterior iliopubic ramus	Mixed	30	no
55	R os coxae	inferior pubic body and anterior ischiopubic ramus	Lytic	30	no
56	L femur	femoral neck, superior three quarters of greater trochanter, quadrate tubercle, intertrochanteric crest, superior two thirds of lesser trochanter	Mixed	CD	*
57	R femur	anterior femoral head	Lytic	20	no
58	R femur	fovea capitis	Mixed	5	no
59	R femur	anterosuperior femoral neck	Mixed	15	no
60	R femur	posteroinferior femoral neck	Mixed	35	yes, with 61
61	R femur	posteroinferior femoral neck	Lytic	10	yes, with 60

*unknown.

L, left; R, right.

For vertebrae: C, cervical; T, thoracic; L, lumbar.

Type: Lytic, osteolytic/osteoclastic lesions; Blastic, osteoblastic lesions; Mixed, both osteolytic and osteoblastic lesions.

Size: maximum dimension (diameter) of lesion in mm or CD, complete destruction of bone portion(s) affected.

## Results

**Figure 4:**
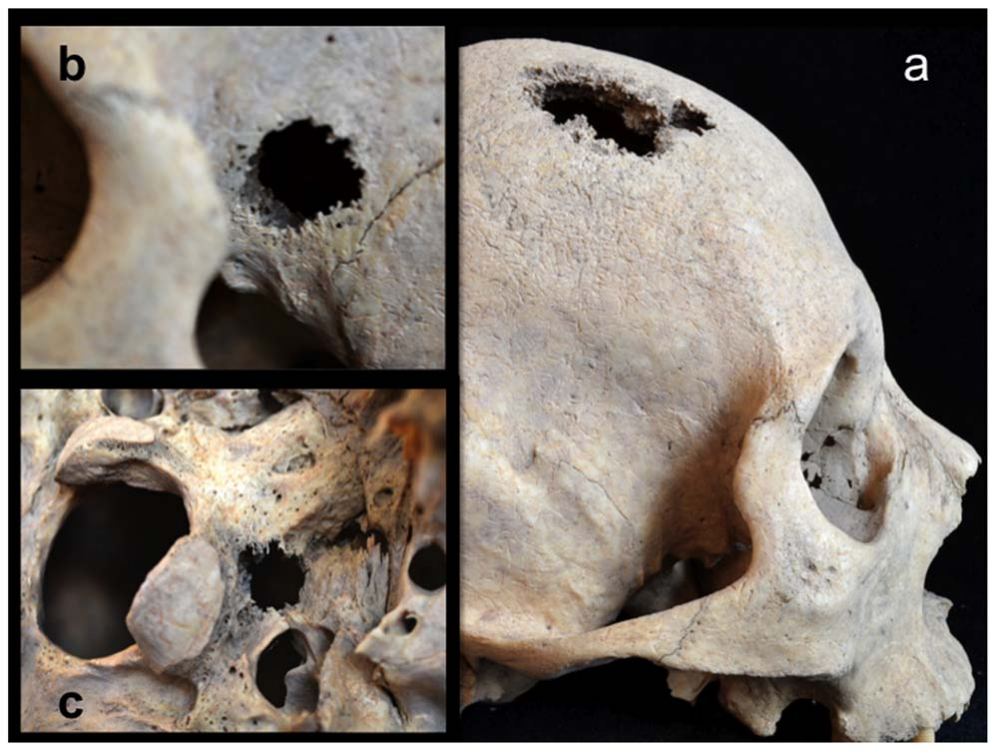
Osteolytic lesions on the cranium; a, coalesced lesions on the frontal bone, right lateral view; b, circular lesion on the left greater wing of the sphenoid (with a small area of reactive woven bone on the anteroinferior margin), left lateral view; c, circular lesion on the left pars lateralis of the occipital bone, inferior view.

**Figure 5:**
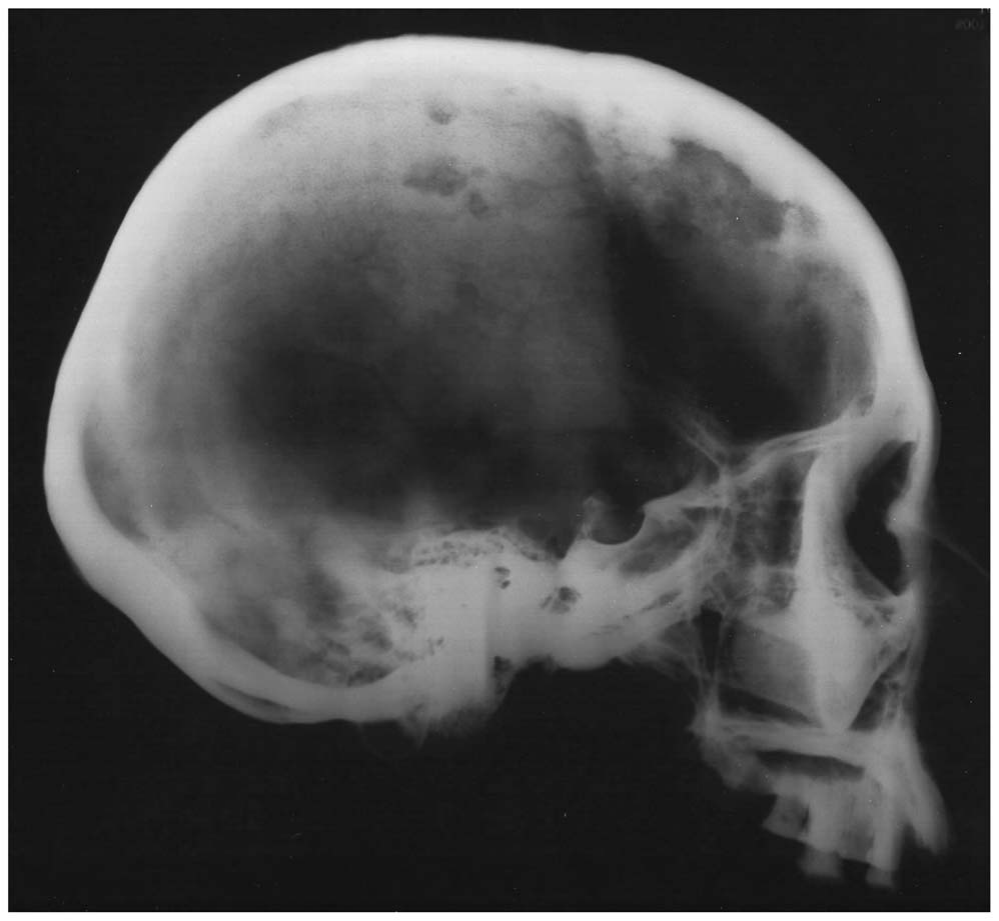
Lateral radiograph of the cranium with at least three areas of diplöic rarification (reduced radio-opacity) on the parietal bones, indicative of additional lesions.

Four perforating lesions measuring between 10 and 35 mm in diameter were observed macroscopically on the cranium of Burial 3: two (coalesced) on the right side of the frontal bone, one on the left greater wing of the sphenoid, and a fourth on the left pars lateralis of the occipital bone (Fig. 4). All four lesions were roughly circular and predominately lytic in nature, exhibiting exposed trabeculae and jagged, irregular margins. In all cases, the diplöic margins exhibited larger diameters than did their adjacent internal and external cortical margins (i.e., residual cortical shells, [43]). Two cranial lesions exhibited small areas of osteoblastic reactive bone (porous new or woven bone) around portions of their peripheries: the anteroinferior external margin of the sphenoid lesion (Fig. 4b) and the internal margin of the larger frontal lesion. A lateral radiograph of the cranium revealed at least three additional lytic foci, represented by areas of reduced radio-opacity, indicative of diplöic rarification [44], on the parietal bones (Fig. 5).

**Figure 6:**
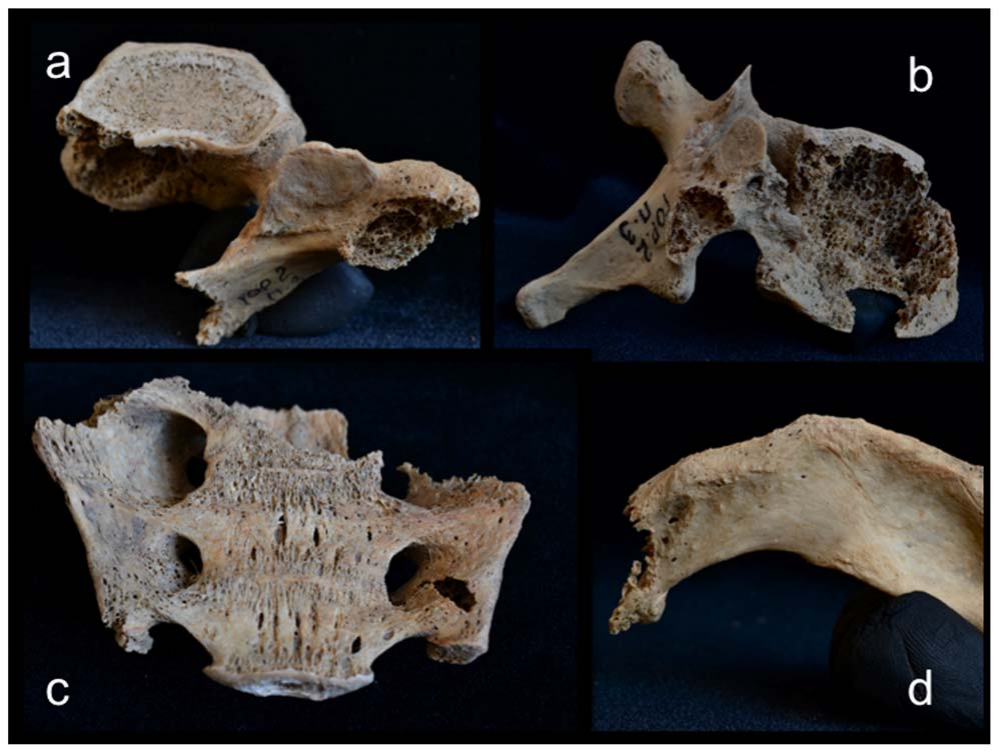
Examples of (predominately) osteolytic lesions on the postcranial axial skeleton; a, cranial thoracic vertebra, superior view; b, caudal thoracic vertebra, right lateral view; c, inferior third of sacrum, anterior view; d, left first rib, superior view.

**Figure 7:**
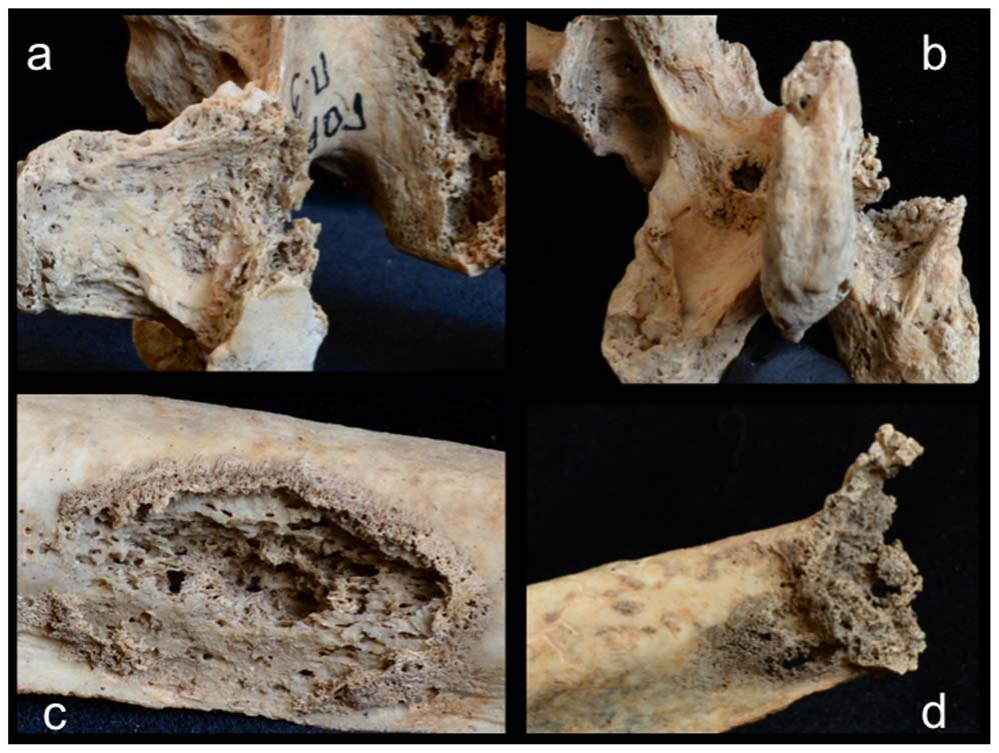
Examples of osteoblastic and mixed (blastic and lytic) lesions on the postcranial axial skeleton; a, T12 vertebra, right lateral view; b, lumbar vertebra, posterior view; c, left rib, pleural view; d, right rib, pleural view.

A total of 34 lesions were documented on the rest of the axial skeleton, just over two thirds (24 out of 34) of which affected the vertebrae and sacrum, with the remaining one third affecting the ribs (Table 1). While vertebral lesions were observed on centra and neural arches more or less equally, costal lesions were concentrated on the proximal (vertebral) portions or midshaft regions, with only one documented on the distal (sternal) end of the bone. Approximately half (18 out of 34) of the postcranial axial lesions were predominately lytic in nature (e.g., Fig. 6), the other half being osteoblastic (bone forming) or mixed osteolytic and osteoblastic (e.g., Fig. 7). Lytic lesions were generally irregular in shape, exhibiting exposed trabeculae and jagged margins that varied widely in size. Some were smaller than 10 mm in diameter (e.g., Figs. 6a and 6b) and others involved large portions of individual elements, such as the complete destruction of the superior two thirds of the sacrum (Fig 6c). Because of missing osseous tissue, it was impossible to measure the size or extent of many larger lesions. Blastic lesions were typically located near, but not immediately adjacent, to lytic lesions and involved the deposition of small nodules of porous woven (or new) bone overlying the existing cortex (e.g., Fig. 7a). Areas of involvement were generally irregular in shape and quite small, not exceeding 15 mm in diameter. Larger and more dramatic osteoblastic responses were always directly associated with areas of osteolysis, being classified as mixed (lytic and blastic) lesions. These lesions, ranging from 10 to 25 mm in diameter on the postcranial axial skeleton, were characterized by solid or, less commonly, spiculated deposits of porous woven bone located either around the periphery of (e.g., Figs. 7b and 7c) or adjacent to (e.g., Fig. 7d) destructive lytic foci. Hook-like projections of nodular woven bone, extending as much as 10 mm beyond the normal cortical surface (e.g., Fig. 7d), were documented on the proximal ends of several ribs.

**Figure 8:**
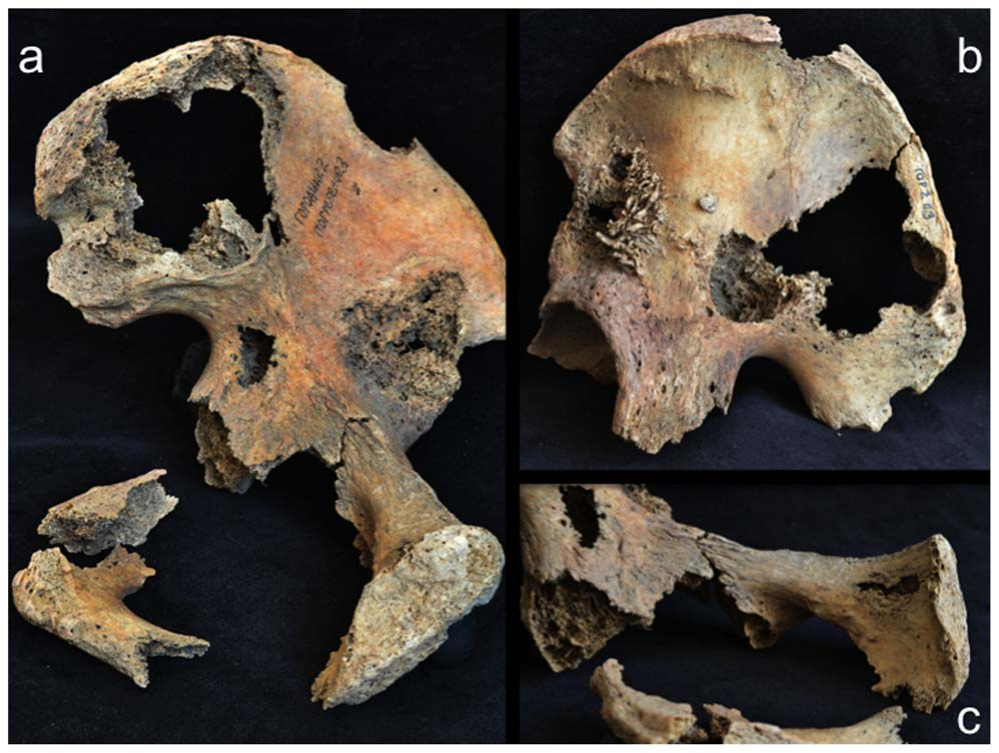
Osteolytic and mixed (blastic and lytic) lesions on the left os coxae; a, medial view of entire element; b, lateral view of ilium; c; posterior view of pubis.

**Figure 9:**
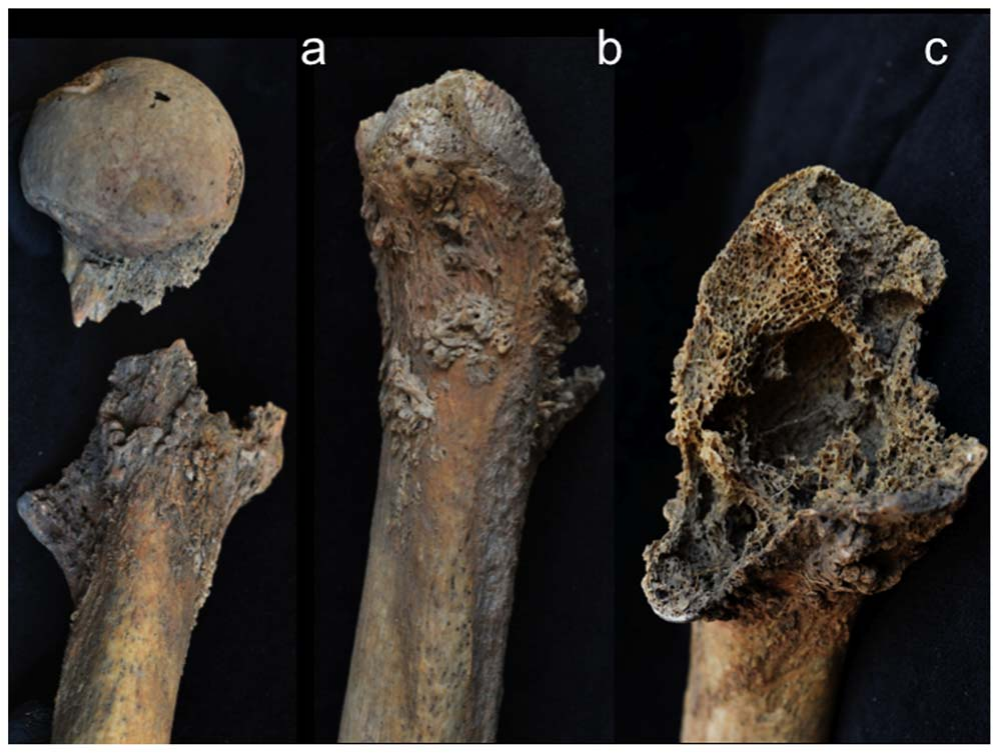
Large mixed (blastic and lytic) lesion completely destroying the left femoral neck and large portions of the trabecular bone within the trochanters; a, anteromedial view; b, lateral view; c, medial view.

**Figure 10:**
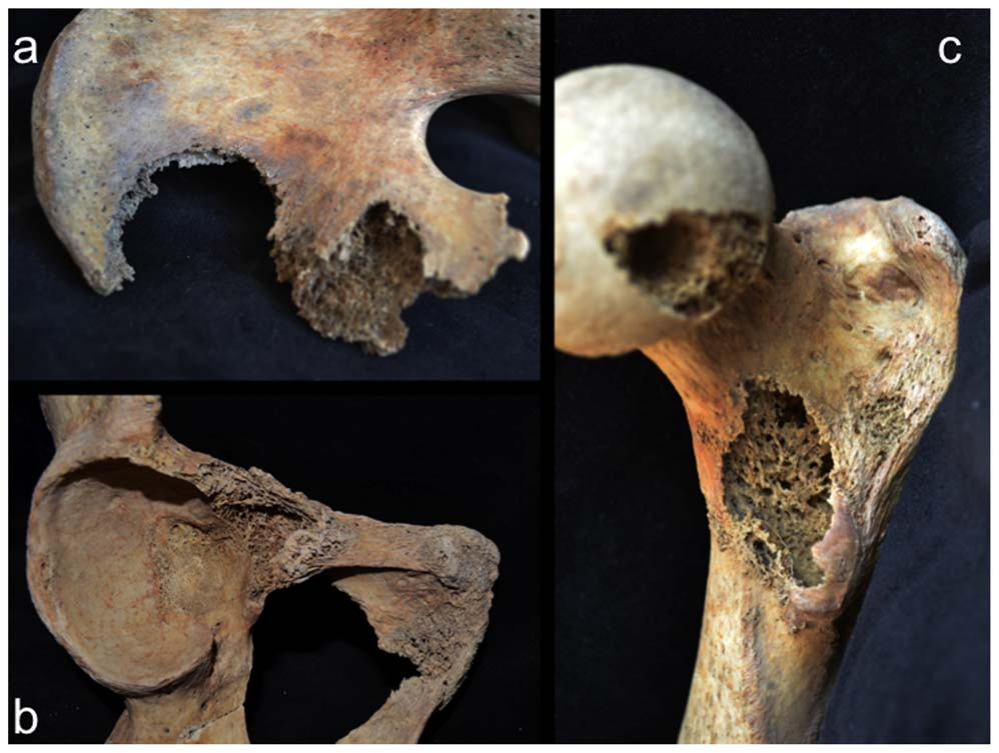
Osteolytic and mixed (blastic and lytic) lesions on the right lower limb elements; a, complete destruction of posterior third of iliac blade, lateral view; b, acetabulum and pubis, anterior view; c, proximal femur, posteromedial view.

**Figure 11:**
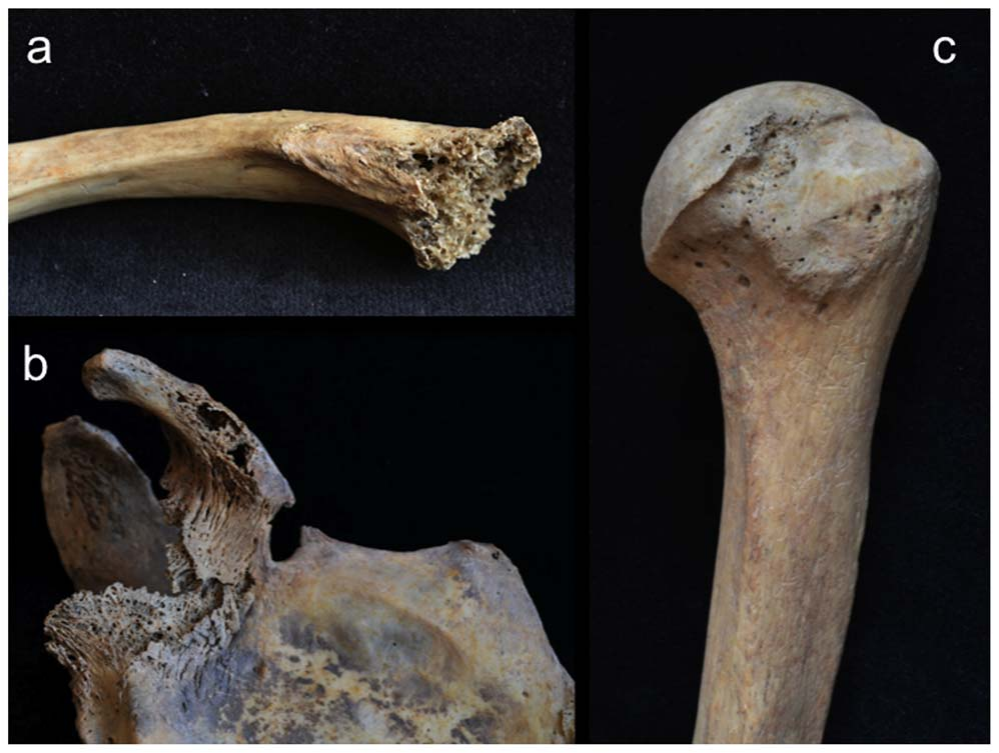
Osteolytic and mixed (blastic and lytic) lesions on the right upper limb elements; a, complete destruction of sternal end of clavicle; b, superior scapula, anterior view; c, proximal humerus, posterior view.

**Figure 12:**
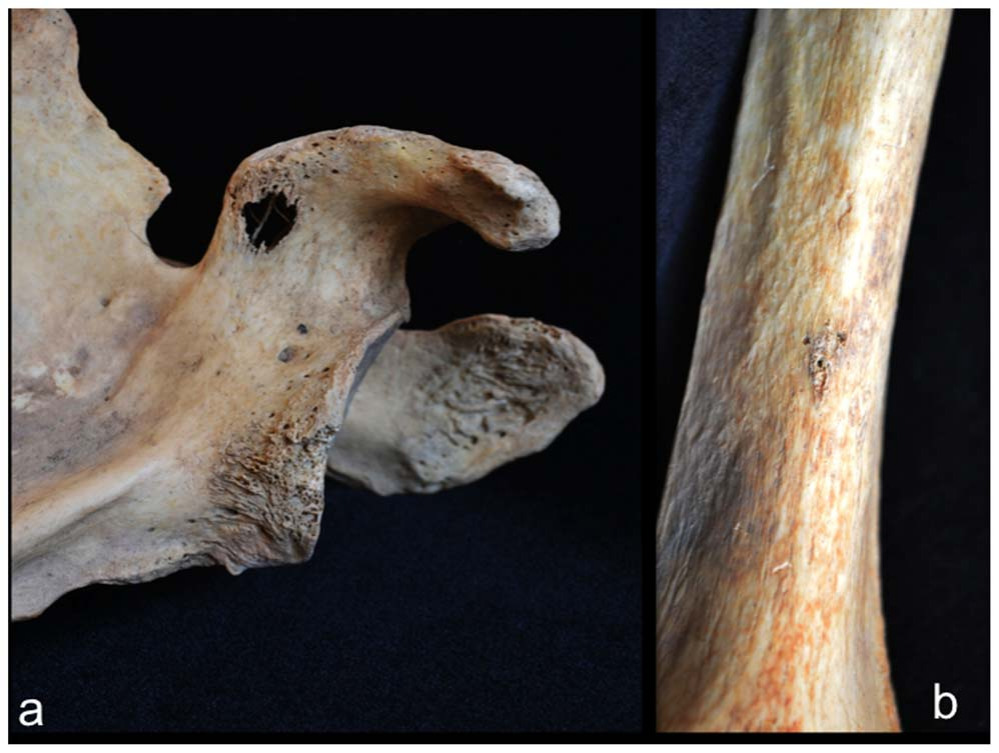
Osteolytic and mixed (blastic and lytic) lesions on the left upper limb elements; a, superolateral scapula, anterior view; b, distal shaft of humerus, anterior view.

**Figure 13:**
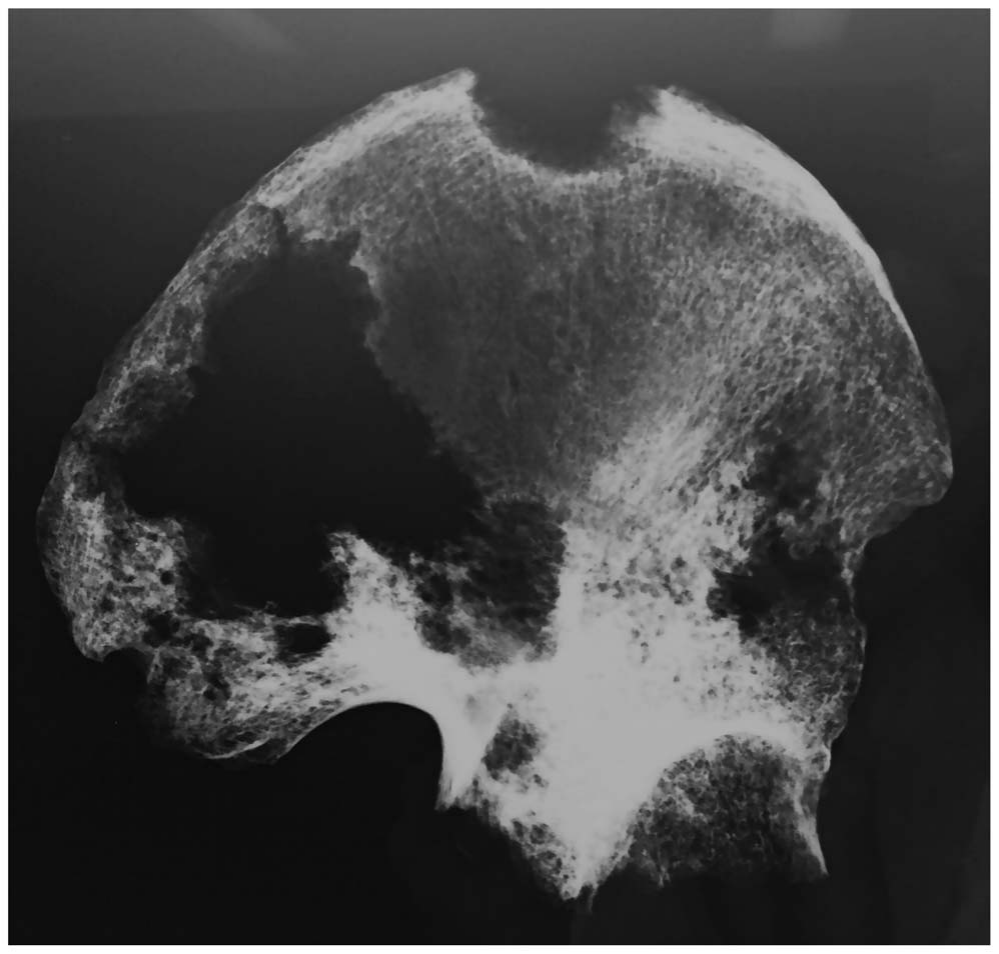
Lateral radiograph of left ilium, with osteolytic lesions indicated by areas of reduced radio-opacity.

Involvement of the appendicular skeleton was limited to the pectoral and pelvic girdles and the proximal portions of the humeri and femora (Fig. 3 and Table 1). In total, 21 lesions were documented, the majority (15 out of 21) of which were observed on the lower limb elements (Table 1). As with the postcranial axial skeleton, about half (12 out of 21) of appendicular lesions were lytic in nature, the remaining half or so being mixed. No purely blastic lesions were documented on this part of the skeleton, as all bone formation was associated with or adjacent to lytic foci (Figs. 8–12). Osteolytic lesions were extensive and highly destructive, many affecting large portions of individual elements such as the left and right ilia (Figs 8a–b and Fig 10a, respectively). While most osteolytic lesions were irregular in shape, several of them (or portions of them) were more or less circular (e.g., Figs 8b, 10a, and 12a). Lesion margins were irregular, with jagged edges and exposed trabeculae, ranging in size from 9 to 66 mm in diameter. In many cases trabecular bone destruction preserved a cortical shell at/around the margins of destructive lesions (e.g., Figs. 8a, 10a, and 10c). Mixed lesions were characterized by nodular (e.g., Fig. 9, 10b, and 11b) or spiculated deposits (e.g., Fig. 8b) of porous woven bone located around the periphery of or adjacent to destructive lytic foci. These lesions ranged from 5 to 41 mm in diameter, many also being highly destructive and affecting large portions of individual elements such as the neck and trochanters of the left femur (Fig. 9) and glenoid cavity of the right scapula (Fig. 11b). Finally, a lateral radiograph of the left ilium (Fig. 13) and anterior radiographs of the right femur and humerus did not reveal any lesions that were not already observed macroscopically.

## Differential Diagnosis

### Postmortem Damage, “Pseudopathology”

Taphonomic processes, especially those associated with microbial organisms, chemical substances, animals, and insects, may damage bone in such a way that mimics the osteolytic process of underlying disease. One example includes a case from Socotran Island that expressed a potentially destructive lesion on the condyles of a left femur—insects were found in the cancellous bone and the damage was associated with boring [45]. Exposure to erosive chemicals such as acid may result in localized destruction of bone that appears similar to the lytic foci of disease processes [46], while microbial organisms tunnel through bone via vascular networks, producing damage that appears similar to pathological lesions [47]. Animal chewing may also produce bone damage that resembles lytic lesions, though is usually accompanied by evidence for gnawing on the epiphyses and longitudinal breaks [46].

Postmortem damage can be differentiated from pathological processes based on several characteristics of bone including the presence of inchoate lesions within cancellous bone, the presence of osteoblastic activity around or near the margins of lesions [48], as well as scanning electron microscopic analysis of Howship's lacunae, irregular trabeculae, and margins of the lesion where osteoclastic activity has occurred [49]. Schultz [49] specifically notes that these methods are useful in cases where destructive lesions are found with little evidence of osteoblastic activity.

Scanning electron microscopic observations were not available for this individual. However, radiographic images revealed destructive lesions within the diplöe of the right parietal that did not involve the inner or outer cortical table (Fig. 5). The left femoral head was broken off at the femoral neck, and lesion formation was possible to observe within the cancellous bone in the trochanteric region (Fig. 9c). In addition, considerable osteoblastic activity was recorded on the anterior surface of the left femoral neck, extending distally onto the proximal portion of the diaphysis (Fig. 9a–b) and on the anterior portion of the external, left ilium (Fig. 8). Evidence for gnawing was absent, and longitudinal breaks in the diaphyses of long bones were absent. Taken together, these findings argue that the destructive processes recorded in this individual were associated with an underlying pathological process rather than postmortem taphonomic damage.

### Tuberculosis

Tuberculosis is an infectious disease associated with consuming the milk/meat of infected mammals or inhaling the infectious bacteria, Mycobacterium bovis or Mycobacterium tuberculosis, respectively [48]. The primary loci of infection include the abdomen and lungs, and the bacteria are disseminated to the skeleton via the mesentery and hilar lymph nodes. Lesions associated with tuberculosis involve the vertebrae, pelvis, hands, feet, ribs, and femur [48], [50]–[51]. Lesion morphology includes a combination of blastic and lytic foci. Lytic foci in vertebrae are characterized by smooth borders with evidence of bony formation around lesion margins, and these lesions are concentrated along the vertebral centra [51]. Healing is reported in cases of spinal tuberculosis and manifests as bone deposition and fusion of vertebral elements [52]. Vertebral kyphosis (collapse) is common when more than one vertebral centrum is involved. Periosteal inflammation of ribs is often observed in association with tuberculosis [53]–[54]. Tuberculosis also produces destructive lesions of the cranium that lack bone formation, while lesions of the hands, feet, and pelvis appear as smooth-walled, lytic foci with evidence for bone formation at the margins [48], [51].

The case in question resembles tuberculosis in terms of lesion distribution. However, the lytic lesions in this study have jagged, irregular margins (Figs. 6–10). Vertebral lesions are found on the vertebral centra, neural arches, and spinous processes (Figs. 6–7)—not simply on the centra—and there is no evidence for a loss of mechanical integrity in the vertebral column. Rib lesions are largely lytic (e.g., Fig. 6d and 7d), as are those of the pelvis and femora (Fig. 6c, 8–10). Where bone production is observed, this process is associated with spiculated or nodular woven bone formation peripheral or adjacent to destructive foci (Figs. 7–9, 10b). These characteristics differentiate the lesions observed in this adult male from those associated with tuberculosis.

### Mycotic Disease

Fungal infections (mycotic diseases) are also important diagnostic considerations for the lesions recorded on this individual. Mycotic infections such as blastomycosis and coccidioidomycosis are described in human, canine, and chimpanzee skeletal remains from North America [55]–[60]. The skeletal manifestation of these two diseases includes destructive lesions with smooth borders, sometimes accompanied by the production of new bone within the destructive focus. These pathogens are not, however, endemic to the Cis-Baikal region, and therefore, remain unlikely diagnostic options for the lesions in question [61]–[62].

Cryptococcosis is associated with several species of fungi, though only Cryptococcus neoformans has a geographic distribution that includes the Cis-Baikal region. Cryptococcus neoformans is a fungal organism that is transmitted via airborne spherules that originate in pigeon excrement [63]. The infection occurs through respiratory pathways and dissemination is largely hematogenous. Skeletal lesions are reported in approximately 10 percent of cryptococcosis infections, and the lesions are circumscribed, destructive foci that are localized in nature [64]–[65]. Skeletal lesions of cryptococcosis are frequently misdiagnosed as cancer metastasis, and therefore, careful consideration of this condition is necessary when providing a differential diagnosis for destructive lesions that may resemble neoplastic conditions [63]. Lesions are frequently found on the skull, vertebrae, and bony prominences (tubercles or tuberosities), but do not involve joint surfaces [63]. Reviews of mycotic disease argue for the presence of a “circumferential periosteal belt” which references woven bone production that circumscribes the lesion border but is not found within the lesion [66].

The lesions observed in this individual retain some similarity with mycotic infection, and given pathogen endemism, cryptococcosis should be considered a possible diagnostic option. Specifically, destructive foci that involve the vertebrae and skull are similar in appearance and general distribution to cryptococcosis. However, the lesions in this individual lack circumferential periosteal formation around the lesion margins and have jagged, irregular borders. In addition, the destructive lesions in this case are more diffuse than those associated with mycotic infection. Therefore, mycotic infection, and specifically cryptococcosis, remains an unlikely diagnostic option for these lesions.

### Multiple Myeloma

Multiple myeloma is a neoplastic disease that results in the accumulation of plasma cells within hematopoietic bone marrow, which then inhibits the production of blood cells. Multiple myeloma is associated with lytic lesions that are diffusely distributed throughout the skeleton. Given the hematopoietic origins of the condition, lesions begin within cancellous bone or on the endosteum and move outward, resulting in the eventual destruction of the periosteum [48]. These lesions are small in size, diffusely distributed, lack the presence of a “residual cortical shell” at or around the margins (where cancellous bone is destroyed, but components of the cortex around the lesion margins remain intact), and have smooth borders that lack new bone formation [43], [67]. Cultures derived from bone marrow among patients with multiple myeloma reveal the presence of an osteoclast activating factor [68]. Recent studies found elevated levels of RANK ligand in combination with MIP1-α or IL-6 in high frequencies of patients with multiple myeloma, and experimental results suggest that the relationship between these transcription factors are important to osteoclast proliferation [69]. Destructive lesions associated with multiple myeloma are approximately 20 mm on average [70], with the largest recorded lesions around 30 mm [71]. The most common skeletal structures involved in cases of multiple myeloma include the vertebrae, ribs, long bones (proximal metaphyseal sections), calvarium, pelvis, scapulae and sternum, and yet, lesions are not found on the femoral head [48], [70].

The lesions associated with this individual are similar to those associated with multiple myeloma, especially the presence of diffusely distributed, destructive lesions that began in cancellous bone. The general anatomical distribution of lesions in this individual also prompts a consideration of multiple myeloma as a diagnostic possibility. However, destructive foci were not found on the radius and ulna, while destructive lesions on the left and right femoral heads were recorded (Figs. 9a and 10c). In addition, osteoblastic lesions were found on a number of elements, most notably on the anterior surface of the left femoral neck, extending distally onto the proximal portion of the diaphysis (Fig. 9), and on the anterior portion of the external, left ilium (Fig. 8b). The presence of a “residual cortical shell” at or around lesion borders was also recorded (Figs. 4a, 6a–b, 7a, 8c). Finally, the maximum size of the lesions reported here (65 mm) were considerably larger than those associated with multiple myeloma. These findings argue against multiple myeloma as a diagnostic possibility for the lesions in question.

### Langerhan's cell histiocytosis

Langerhan's cell histiocytosis is a reticuloendothelial disease and is another diagnostic possibility for these lesions. This condition is characterized by the abnormal proliferation of histiocyte cells and increased dissemination of these phagocytic cells throughout the vascular system [48]. Lesions associated with histiocytosis can be focal or diffuse, most frequently involving the skull, vertebrae, ribs, and less frequently, long bones. The lesions appear as destructive, round or oval masses, and have a beveled edge around the external border of the lytic focus, with slight bone formation [48]. Demographically speaking, this condition is focused on immature individuals, as 80 percent of all cases occur before 30 years of age, while 50 percent of all cases occur before 10 years of age [72]. Survival beyond adolescence is rare [48]. One set of lytic lesions in a juvenile, aged 5.0 to 10.0 years, from Pre-Angkorian Cambodia (ca. 2000 yBP) is illustrative of this condition [73]: the individual has multiple lytic lesions around the cranium. The lesions are circular/ovoid in shape and have evidence of slight bone formation around the lesion margins. The external surface of the lesion borders are beveled, while the demographic profile of the individual from Cambodia is consistent with Langerhan's cell histiocytosis.

The morphological appearance and distribution of lesions described in the individual from Cis-Baikal necessitate a consideration of Langerhan's cell histiocytosis as a possible diagnostic option. However, the lesions in this case are differentiated from those of Langerhan's cell histiocytosis based on irregular borders, lack of beveling at the lesion margins, and an anatomical distribution on long bones. Furthermore, the age of this individual (35.0 to 45.0 years) suggests Langerhan's cell histiocytosis is a less likely diagnostic option.

### Metastatic Carcinoma

Meastatic carcinoma refers to a series of malignant tumors that arise in epithelial tissue and are hematogenously disseminated to the skeletal system [48]. These tumors may arise in any number of soft-tissue structures including the lung, breast, prostate, or kidneys. Microscopic evaluation of soft-tissue is necessary to provide a diagnosis that includes the primary site of tumor growth when metastatic carcinoma is documented in human skeletal remains [48]. Skeletal lesions associated with metastatic carcinoma are diffusely distributed, with the most frequent sites for bone destruction including the vertebrae, ribs, pelvis, and proximal femur followed by the proximal humerus, skull, distal femur, and clavicle, while occasional sites include the proximal tibia, distal humerus, and mandible [74]. Distribution of lytic lesions on the humerus most frequently involve the medullary and cortical areas of the shaft, while those of the femur are most often distributed on the femoral head, extending distally to the trochanteric region [74]. Lesions associated with metastatic carcinoma in individuals who did not receive clinical therapy are primarily destructive, range in size from 3 to 47 mm, and may be independent or coalescent in nature [44]. These lesions begin in the hematopoietic regions of cancellous bone, and proceed outward, destroying the outer layer of cortical bone [43]–[44], [48], [74]. Lytic lesions associated with cancer metastasis frequently preserve a “residual cortical shell” around the margins [43]. Spiculated bone formation is often observed in areas near or around lytic foci, but is not found around the borders of lesions [48].

The anatomical distribution of lesions in this individual includes bones most commonly involved by metastatic carcinoma, including the vertebrae, femur, skull, ribs and sternum, pelvis, and shoulder girdle (Fig. 3). Lytic foci on the femur involve the head and trochanteric regions. A considerable number of inchoate lesions were found in the cancellous bone of the trochanteric region of the left femur (the femur was broken at the neck and it was possible to see inside this bone; Fig. 9c), while a large destructive process was also observed at the lesser trochanter of the right femur (Fig. 10c). Radiographic and macroscopic analysis reveals that the lytic foci began in cancellous bone (Fig. 5, 6b, 8a–b, 9c, 10c, 12a) and there is evidence for the preservation of a “residual cortical shell” around the margins of many lesions (Figs. 8a, 10a, and 10c). The size of destructive lesions is variable, ranging between 5 to 66 mm, and there is evidence for both focal and coalescent lesion formation. Spiculated bone formation was observed on the anterior surface of the left femoral neck, extending distally onto the proximal portion of the diaphysis (Fig. 9), and on the anterior portion of the external, left ilium (Fig. 8b). No aspects of the lesion morphology or anatomical distribution in this case argue against a diagnosis of metastatic carcinoma. On this basis, metastatic carcinoma remains the most likely diagnostic option for these lesions.

## Discussion

The lesions described here represent one of the oldest probable cases of cancer metastasis worldwide and the earliest identified from Northeast Asia. That this individual was a member of an ancient hunter-gatherer population (see above) is equally noteworthy, given that the vast majority of metastatic carcinoma cases documented in the paleopathological literature—even very early ones (e.g., [3], [10]–[11])—represent agricultural groups. It is not possible to reconstruct the specific etiology of this particular malignancy, considering the multitude of genetic and environmental factors that have been linked to cancer in modern humans, but we can make some basic inferences regarding its possible origin(s) and progression. Of those carcinomas that frequently spread to bone (kidney, lung, breast, gastrointestinal, thyroid, uterus, and ovary), breast and lung carcinomas are the most likely to produce mixed osteolytic and osteoblastic lesions [75]–[76], such as those documented here. However, because breast cancer is extremely rare in males (e.g., [77]), lung cancer is the more likely diagnostic option of the two. On the other hand, lesion distribution, particularly the extensive involvement of the pelvis and lumbar vertebrae, may suggest prostate cancer. Carcinoma of the prostate most often produces osteoblastic lesions, but osteolytic foci are also reported [48]. Furthermore, unlike other carcinomas, those of the prostate tend to affect the pelvis and lumbar vertebrae disproportionately, probably via direct periprostatic-prespinal venous communication [78]. The extensive (and disproportionate) involvement of these elements is documented above and illustrated in Figures 3, 6a, 8, and 10. Thus, while lesion appearance supports a diagnosis of lung cancer, lesion distribution may also suggest prostate cancer.

It is impossible to definitively identify the primary site of the carcinoma in this case, be it the lung, prostate, or another tissue, but it is clear that the disease had progressed considerably, metastasizing far beyond its original location in the body, and that it contributed to the death of this individual. The progressive state of the disease, and its bony involvement in particular, would have caused severe pain and even disability during the last weeks of life [76]. A discussion about care and compassion among the ancient foragers of the Cis-Baikal is beyond the scope of paleopathological research. It is, however, interesting to note the context in which this individual was interred. Circular pits and tightly-flexed burials were unusual in the Early Bronze Age of Cis-Baikal; most contemporaneous burials were situated in elongated pits and in extended and supine positions [79]–[80]. In addition, the rich grave goods associated with Burial 3, specifically the ornamental bone tubule and the bone spoon with a carved serpent handle, suggest something unique about this individual and his role in the community. Most Early Bronze Age males were buried with items such as hunting and fishing paraphernalia [79]–[80]. In fact, both Early Bronze Age burials excavated from Gorodishche II were unique in their body positioning (the other being seated) and rich grave goods (the other containing a wild boar fang pendant, a bronze clasp, and a partially worked pendant made of rare white nephrite). The location of the cemetery was also unusual in that it was situated only 2.5 km from the much larger (n = 67) Late Neolithic-Early Bronze Age cemetery of Ust'-Ida I. All of this supports the interpretation that the mortuary treatment of Burial 3 is consistent with an identity that was distinct from the general community, possibly owing to—or, perhaps, in spite of—health status or the circumstances surrounding death.

While this case is the most complete and diagnostically sound example of cancer from the Cis-Baikal, it is not the only one. In fact, two other possible cases of malignant neoplasia—one metastatic carcinoma and one multiple myeloma—have been documented in the region. A male, aged 30–35 years (Burial 6.1), from the nearby Late Neolithic-Early Bronze Age cemetery of Ust'-Ida I exhibited a large (2.5-3 cm in diameter) lytic lesion on the right frontal bone [81]. The lesion was solitary and predominately lytic in nature, with exposed trabeculae and jagged, irregular margins consistent with metastatic carcinoma (Fig. S2). The individual was directly radiocarbon dated to 5814–5659 years BP (TO-10312: 4960±90 cal. BP [82]). The second example was an older adult (50+ years of age at death) male (Burial 49.1) from the Early Bronze Age cemetery of Khuzhir-Nuge XIV, located in the ‘Little Sea’ region of the Cis-Baikal (Fig. 1). While poorly preserved, the skeleton exhibited numerous small (0.5–1 cm in diameter) perforating lytic lesions on the femora, tibiae, fibulae, and cranial vault, those on the fragmented vault coalescing into larger lesions. There was no evidence of osteoblastic activity adjacent to or associated with the bony destruction. In addition, the deformed right femoral shaft—the cortex representing an expanded bony shell and probable initial site of the disease (Figure S3)—suggests that these lesions are consistent with multiple myeloma [81], [83]. The individual was directly radiocarbon dated to 4634–4484 years BP (TO-10312: 4030± 60 cal. BP [82]).

The case presented here is also of considerable interest to individuals studying the history and evolution of neoplastic diseases. The Late-Neolithic-Early Bronze Age people from Gorodishche II and other Cis-Baikal cemetery sites were members of a broad-spectrum foraging economy. As mentioned above, cancer metastasis is rarely reported as a probable diagnostic option for pathological lesions in hunter-gatherer skeletal samples (for examples see: [6], [9], [84]). The relative dearth of these cases has prompted some researchers to suggest that metastatic carcinoma was rare in pre-industrial populations because of shorter life expectancies (e.g., [15], [20], [85]). This statement is, however, not based on the factual reality of hunter-gatherer demography. Recent studies demonstrate that life expectancy in modern hunter-gatherers is similar to Western human populations [86]. The interpretation that past hunter-gatherers and Paleolithic humans had reduced life expectancies is also based on faulty assumptions and questionable demographic reconstructions. For example, human life expectancy at birth is reduced in association with epidemiological cycles between the Mesolithic and late Middle Ages in Europe, suggesting that the human tendency towards longevity did not increase over time, but instead, was contingent upon local environmental conditions [87]. Other explorations of mortality demonstrate that prehistoric foragers had life expectancies that fall within the range of modern foragers, and that circumstances where these life expectancies are reduced may be associated with selective bias in cemetery usage [88]–[90]. These findings suggest that the scarcity of metastatic carcinoma among prehistoric foragers is not due to demographic underrepresentation of older individuals.

Alternatively, cases of metastatic carcinoma may be relatively rare in prehistory due to misdiagnoses or the failure of many observers to record the lesions associated with cancer metastasis [9], [45]. Skeletal lesions attributable to metasatic carcinoma often resemble other diseases in terms of morphology and anatomical distribution [43]–[45], [48]. On this basis, it is possible that misdiagnoses have reduced the number of cases observed among hunter-gatherers. However, even more likely is the possibility that the lesions associated with this disease are infrequently recorded. Metastatic carcinoma produces destructive lesions that are often mistaken for postmortem damage, particularly when preservation is poor, and this similarity likely causes a number of specialists to miss potential cases in the bioarchaeological record [45]. As researchers become more familiar with the skeletal manifestations of metastatic carcinoma, the number of cases identified by bioarchaeological research is likely to increase. For example, the identification of diseases such as scurvy increased following an improved familiarity with the skeletal manifestations of the condition [91].

## Conclusions

This case is noteworthy for several reasons, not in the least because it represents one of the oldest examples of metastatic carcinoma documented in the world, and the earliest recorded in Northeast Asia. That the afflicted individual was a member of a hunter-gatherer population—and that several other possible cases of malignancy were observed in the same population—challenges long held assumptions regarding the demographic underrepresentation of older individuals among prehistoric foragers. In fact, it is more likely that misdiagnoses and/or inadequate documentation, the latter a reflection of skeletal preservation, are at least partially responsible for the perceived rarity of cancer in antiquity. An increased awareness among scholars of the skeletal manifestations of cancer metastases is essential in order to more fully understand the temporal and spatial distribution of neoplasia and to more clearly reconstruct its history and evolution.

## Supporting Information

Figure S1 **One of the rich grave goods associated with Burial 3: a unique bone spoon with a carved winding serpent handle.**
(TIF)

Figure S2 **Ust'-Ida I, Burial 6, male aged 30–35 years with possible metastatic carcinoma: sclerotic lytic lesion with jagged irregular edges on left frontal bone.**
(TIF)

Figure S3 **Khuzhir-Nuge XIV, Burial 49, male aged 50+ years with possible multiple myeloma.** A, coalesced lytic lesions on the left cranial vault with smooth borders lacking bone formation; B, left (right) and right (left) femora with small lytic lesions on the diaphyseal cortices (no evidence of osteoblastic activity) and abnormal shape (expanded bony shell) of right femoral cortex.
(TIF)
