# Supplementary figures and images for: Paleopathological Description and Diagnosis of Metastatic Carcinoma in an Early Bronze Age (4588+34 Cal. BP) Forager from the Cis-Baikal Region of Eastern Siberia
Source: PLoS One. 2014 Dec 3;9(12):e113919. doi: 10.1371/journal.pone.0113919 (PMC4254749; doi:10.1371/journal.pone.0113919)

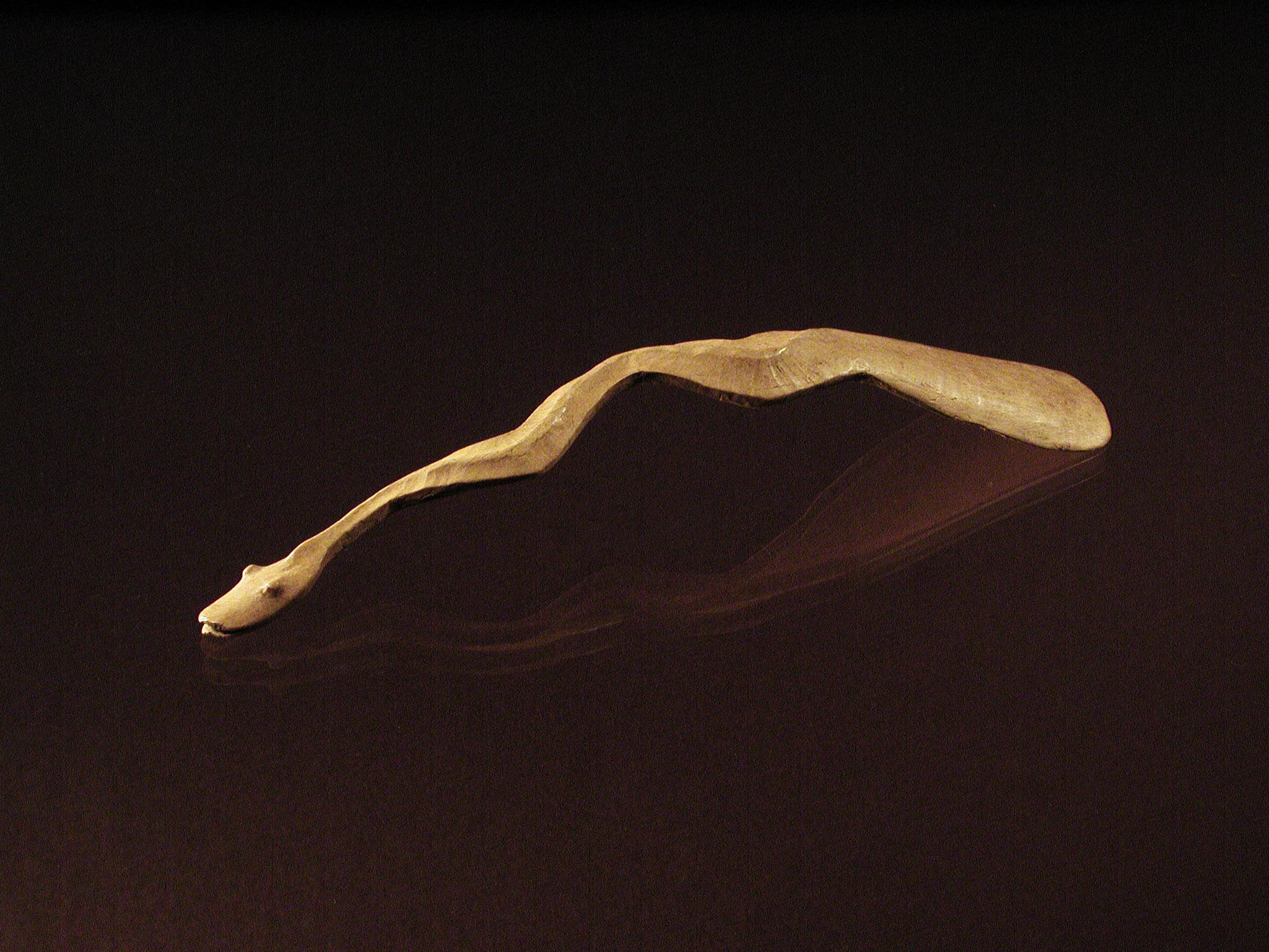

Supplement: Figure S1 — One of the rich grave goods associated with Burial 3: a unique bone spoon with a carved winding serpent handle. (TIF) [file pone.0113919.s001.tif]

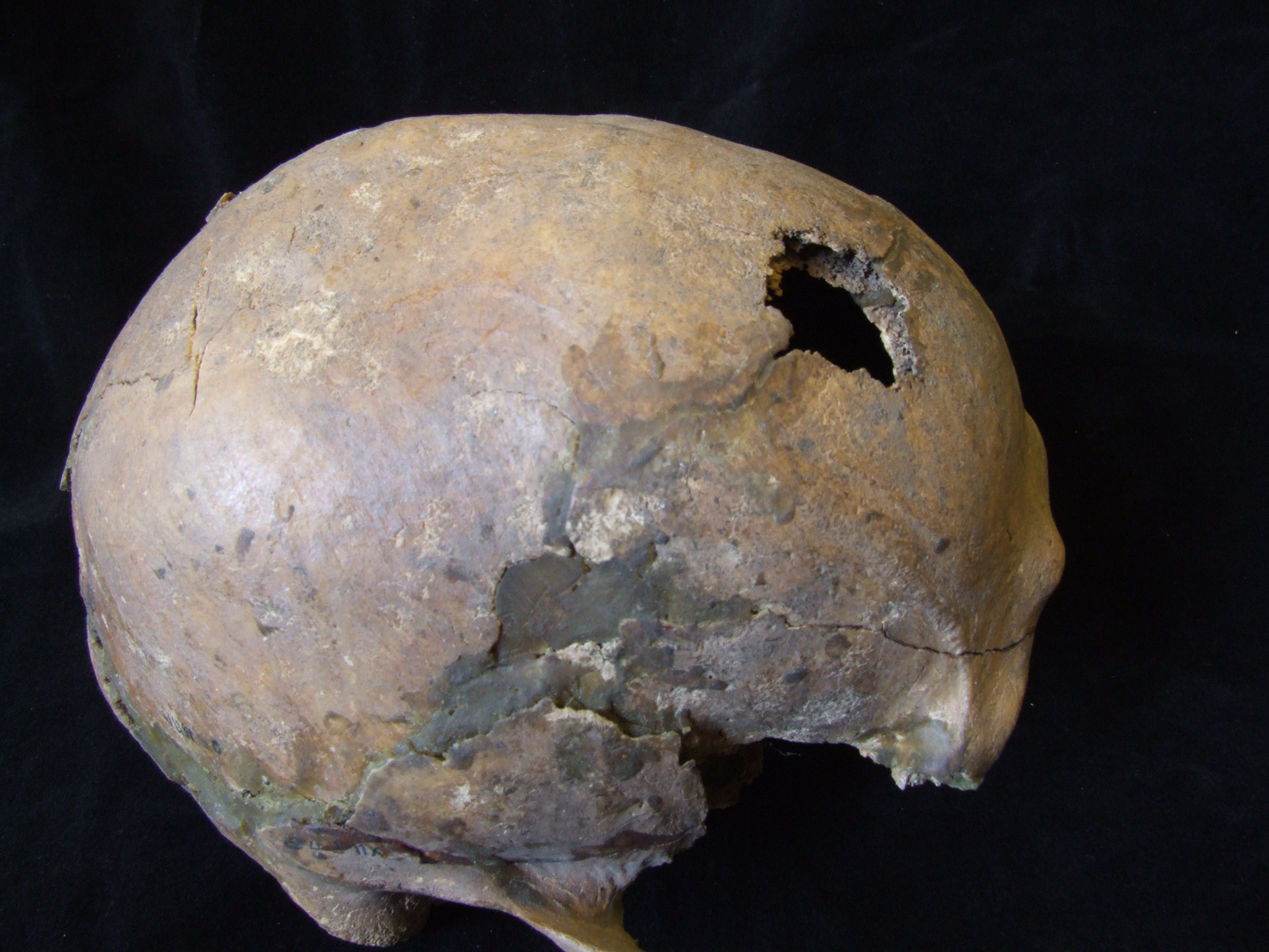

Supplement: Figure S2 — Ust'-Ida I, Burial 6, male aged 30–35 years with possible metastatic carcinoma: sclerotic lytic lesion with jagged irregular edges on left frontal bone. (TIF) [file pone.0113919.s002.tif]

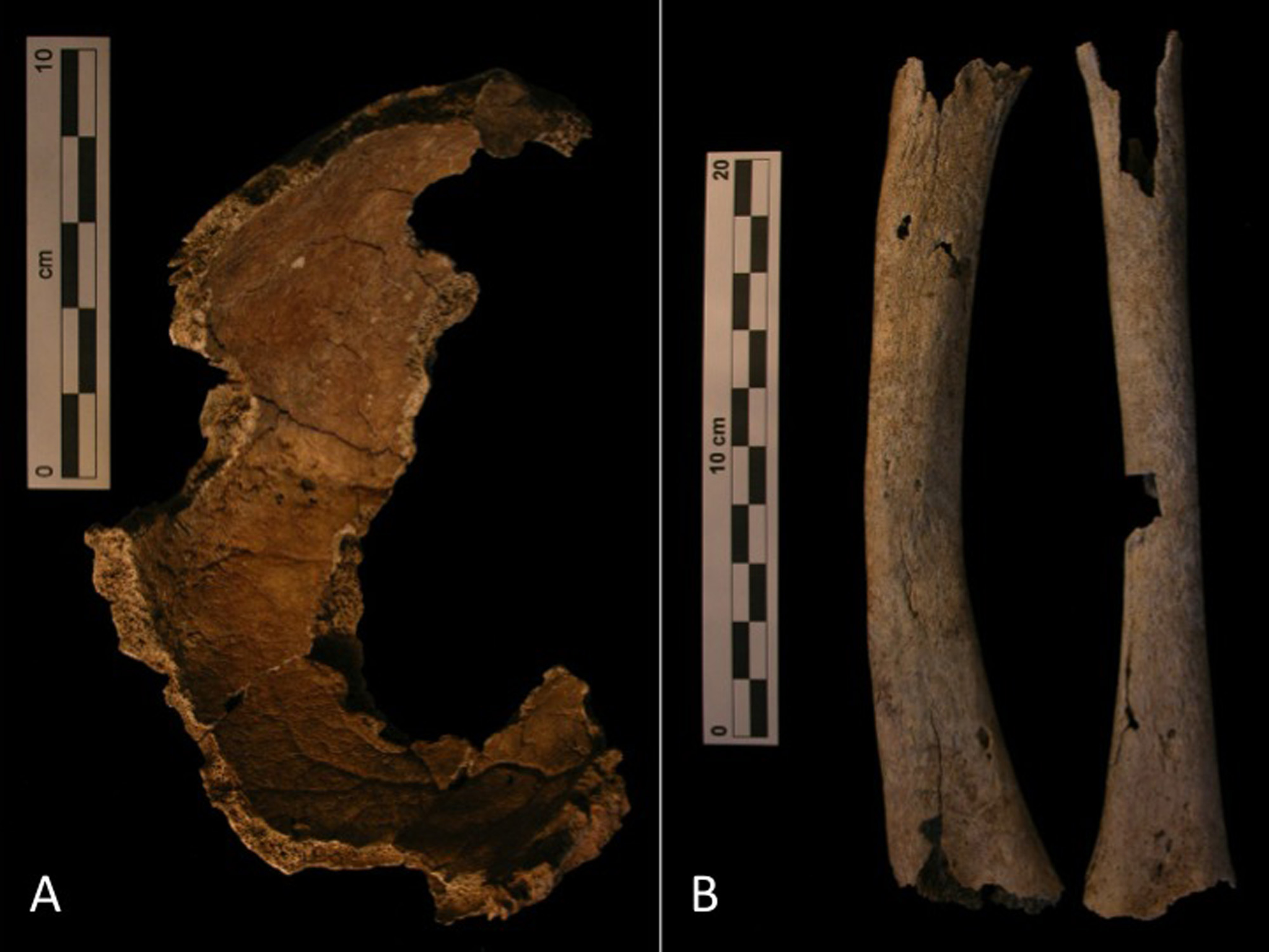

Supplement: Figure S3 — Khuzhir-Nuge XIV, Burial 49, male aged 50+ years with possible multiple myeloma. A, coalesced lytic lesions on the left cranial vault with smooth borders lacking bone formation; B, left (right) and right (left) femora with small lytic lesions on the diaphyseal cortices (no evidence of osteoblastic activity) and abnormal shape (expanded bony shell) of right femoral cortex. (TIF) [file pone.0113919.s003.tif]
